# Wastewater Biofilm Photosynthesis in Photobioreactors

**DOI:** 10.3390/microorganisms7080252

**Published:** 2019-08-10

**Authors:** Antonella Guzzon, Francesca Di Pippo, Roberta Congestri

**Affiliations:** 1 LBA-Laboratory of Biology of Algae, Department of Biology, University of Rome “Tor Vergata”, 00133 Rome, Italy; 2 CNR-IRSA, National Research Council, Water Research Institute, Area della Ricerca di Roma 1, 00010 Montelibretti (RM), Italy

**Keywords:** phototrophic biofilm, wastewater, photosynthesis, photobioreactor, Pulse Amplitude Modulated fluorescence, biofilm biomass

## Abstract

Photosynthetic performance of algal-bacterial biofilms from an Italian wastewater treatment plant was studied in a flow-lane photobioreactor at different irradiances, temperatures, and flow regime to evaluate the effects of these environmental parameters on biofilms’ functioning, in view of application of these communities in wastewater biological treatment. Pulse amplitude modulated fluorescence was used to estimate the effective quantum yield of PSII (ΔF/F_m_’) of the light-acclimated biofilms and to perform rapid light curves (RLCs) for the determination of the photosynthetic parameters (rel.ETR_max_, α, I_k_). Chl *a*, ash free dry weight (AFDW), and dry weight (DW) were measured to assess phototrophic and whole biofilm biomass development over time. From the analysis of photosynthetic parameter variation with light intensity, temperature and flow rate, it was possible to identify the set of experimental values favoring biofilm photosynthetic activity. Biomass increased over time, especially at the highest irradiances, where substrata were fastly colonized and mature biofilms developed at all temperatures and flow conditions tested.

## 1. Introduction

Biological treatment is an important and integral part of wastewater treatment (WWT) procedures. Currently, the activated sludge process is the most commonly applied biological WWT technology. The activated sludge treatment utilizes dense bacterial suspended communities, forming biological flocs, to degrade organic material under aerobic conditions [1]. Although this technology provides satisfactory pollutant removal, it requires complex and multistep recycles of activated sludge to meet the limits imposed by environmental regulations. The overall increase of process costs, complexity, and energy input has led, in the last years, to explore more sustainable approaches [2,3]. Thanks to the synergistic relationship between photosynthetic and heterotrophic microorganisms, the use of microalgal-bacterial consortia in WWT is now considered a cost-effective, efficient, and sustainable alternative to conventional activated sludge treatment, especially for municipal wastewaters, due to the potential for cost-free oxygenation, efficient nutrient removal, and carbon dioxide sequestration provided by microalgae metabolism [4,5]. In this way, microalgal-bacterial combinations have been employed for treating secondary wastewater effluents in the form of suspensions in the enhanced pond and wetland (EPW) system, a multiple step process including high-rate algal ponds (HRAPs) [6], and gravity algal harvesters (AHs) [7]. Alternatively, microalgal-bacterial consortia have been grown as attached communities in algal turf scrubbers (ATSs) [8], coupled to constructed wetlands [9] or as microalgal-integrated fixed film activated sludge (MAIFAS), an advanced biological WWT process that integrates distinct algal and nitrifying biofilm carriers within conventional activated sludge [10]. 

Although the complex interactions between microalgae and bacteria in WWT are not yet fully elucidated, outputs of many studies conducted to date have suggested that microalgae, through photosynthesis, provide oxygen to heterotrophic bacteria to reduce chemical oxygen demand (COD). On the other side, bacteria release carbon dioxide and mineral nutrients from respiration and organic matter degradation which are efficiently converted into biomass by microalgae when exposed to solar light in outdoor systems [2,4,11,12]. Simultaneously, the exchange of metabolites results in an overall increase in biomass productivity and hence, pollutant removal efficiency. Moreover, WWT based on microalgae-bacterial consortia has the added benefits to allow for nutrient recover and biomass recycle into valued compounds as fertilizers, growth enhancers, aquaculture feeds, and/or bioenergy, also implementing a biorefinery approach of the biomass produced starting from wastewaters and up-cycling of the effluent water [13,14,15]. The use of municipal wastewaters as a source of nitrogen and phosphorus to feed the algae is also important for reducing the cost of culturing biomass destined for commercial purposes, such the extraction of biocompounds [16]. In this context, the use of phototrophic biofilms, attached matrix-enclosed microbial communities comprising both phototrophs and chemotrophs, can offer an additional advantage over HRAPs, in terms of time and cost savings involved in biomass accumulation and harvesting. Biofilm indeed is easily removed by scraping it off from the growth support. Moreover, an effluent with a lower total suspended solids concentration is produced with respect to suspended growth systems [12,17,18,19,20]. Despite the favorable outlook of wastewater fed biomass production, routine large-scale application of benthic photosynthetic communities in WWT treatment systems is not efficiently exploited, with much effort put on research only at the laboratory and/or pilot scale [2,21]. Phototrophic biofilm efficiency in wastewater treatment depends on biomass development and physiology, including photosynthetic activity [22]. Although studies have addressed the potential of nutrient removal from wastewaters [23,24,25,26] and the intracellular accumulation by aquatic phototrophic biofilms [27], few have investigated photosynthetic performance and evaluated the environmental factors influencing the process, i.e., geographical location, temperature and oxygen accumulation, light availability, light–dark cycle, as well as pH variation with photosynthesis. 

Therefore, understanding the overall community photosynthesis and photoacclimation capability is pivotal to the exploitation of phototrophic microbial consortia in biological treatment of wastewaters. 

Different approaches have been applied to measure photosynthesis of aquatic phototrophic biofilms, such as oxygen microelectrodes, photosynthetic chambers in combination with oxygen probes, and pulse amplitude modulated (PAM) fluorimetry [28,29,30,31,32,33,34,35]. The fluorescence method is advantageous for estimating the rates of photosynthesis for intact biofilms being rapid and non-destructive, as measurements are performed in a shorter time than conventional methods and without the need to remove the phototrophs from the substrata [32]. Moreover, the parameters measured allow insight into key aspects of photosynthetic light capture and electron transport [36].

Accordingly, the aim of this study was to investigate the photosynthetic behavior, biomass development, and production of phototrophic biofilms collected from an Italian WWT plant and cultured in a flow-lane incubator system at different abiotic conditions. Species composition, matrix characterization and nutrient removal capability of these autotrophic-heterotrophic cultured biofilms have been previously investigated [27,37,38,39,40]. Therefore, the present work provides additional useful insights into phototrophic biofilms functioning, thus increasing the knowledge of how these complex autotrophic–heterotrophic consortia behave at different environmental factors, in view of their exploitation in wastewater bioremediation.

## 2. Materials and Methods 

### 2.1. Biofilm Sampling and Setup of Biofilm Cultures

Biofilm samples were collected by scraping off the overflow system of the sedimentation tank (ST) of an Italian wastewater treatment plant (WWTP) located in Fiumicino (Rome, Italy). Taxonomic assessment of in situ consortia can be found in [41,42,43,44,45]. The plant is designed to serve the Airport “Leonardo da Vinci” and receives municipal wastewater with an estimated capacity of 6000 m^3^ sewage per day. Physico-chemical characteristics of the plant have been previously described [41,46].

After sampling, ST biofilm biomass was subjected to a pre-treatment to obtain safe-handling and homogeneous inocula according to [43]. Briefly, scrapings were washed in 2% sodium hypochlorite for 15 min to eliminate macro- and microzoobenthos and then rinsed in BG11 medium [47] modified for ensuring balanced algal growth by adding vitamins and silicates for diatoms. Moreover, NO3^−^ and PO4^−^ concentration was modified to approximate wastewater levels. Thereafter, biofilms were homogenized and frozen at −20 °C for 24 h to remove grazers [43]. To start the cultures, 100 mL of cell suspension was made up to 4 L with the modified BG11 and filtered through a 300 µm net.

The resulting mixture was inoculated in a semi-continuous flow-lane incubator system (PBI) [48].

**Figure 1:**
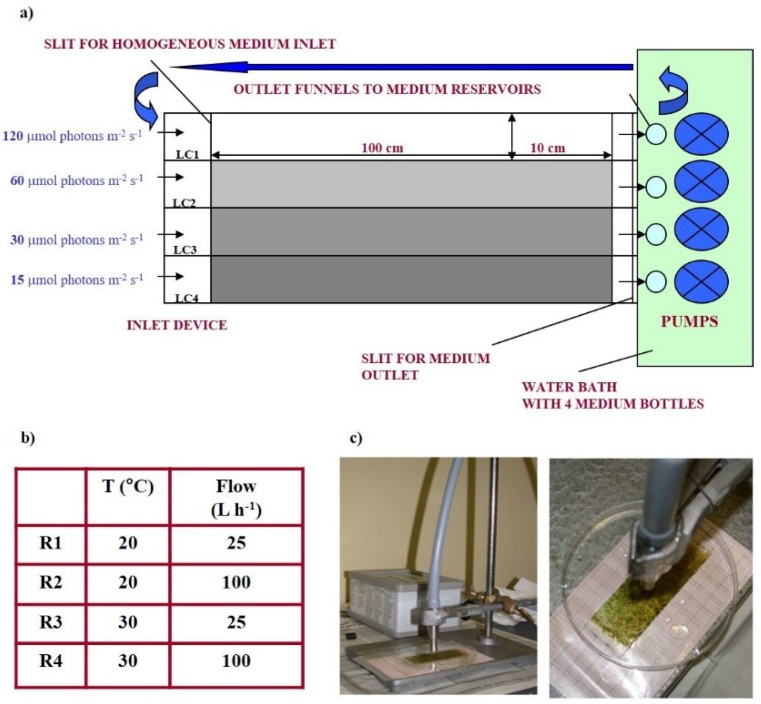
Schematic view of the prototype incubator: In and outlet, light chambers with separator walls for medium guidance, illuminated at four different irradiances (**a**). Experimental conditions of the four runs performed (**b**). MINI-PAM used to perform fluorescence measurements on biofilms colonizing the slides in the incubator with the fiberoptic positioned above biofilm surface (**c**).

PBI contained four separate light chambers (LCs) (120 × 10 cm each) (Figure 1a).

Medium flowed above polycarbonate slides (76 × 25 × 1), used as substratum for biofilm growth, and was changed at regular intervals (twice per week). Flow rate was valve-regulated. “True light” (T-8, Auralight, Sweden) lamps were used as the light source. 

Biofilms were cultured at four photon flux densities (PFDs): 120 μmol photon m^−2^ s^−1^ (LC1), 60 µmol photon m^−2^ s^−1^ (LC2), 30 µmol photon m^−2^ s^−1^ (LC3), 15 µmol photon m^−2^ s^−1^ (LC4). Two temperatures (20 °C and 30 °C) and two flow conditions (25 L h^−1^ and 100 L h^−1^) were tested. These four incubator experiments were referred to as “Runs” (Figure 1b). Transmittance was measured in each chamber by nine subsurface light sensors located under the growth surface. Growth of the biofilm could be accurately tracked by the reduction in light transmittance. The incubator design was previously described [48]. All light and temperature sensors were connected to a computer through a serial port for continuous data monitoring and acquisition [48]. 

Biofilms were sampled at three stages of development as function of biofilm light absorbance values (Absorbance = 100%—percent transmitted light value): 

(1) Initial (in), when the average value of absorption was 10%; 

(2) Active (ac), when mean value was 50%; 

(3) Mature (ma), at 90–95%. 

Each run lasted ~30 days. On the last day, biofilm was collected without regard of light absorption and labelled as last sampling (ls). 

### 2.2. PAM Measurements of Photosynthetic Parameters

Photosynthetic parameters of cultured biofilms were assessed at each sampling stage using a miniaturized pulse amplitude modulated (Mini-PAM) fluorometer, coupled to WinControl Software (Walz GmbH, Effeltrich, Germany) for computer control and data analysis. In the Mini-PAM, fluorescence was excited by pulses (3 μs width) of measuring red light from a light-emitting-diode (LED) with the peak excitation band at 650 nm. Fluorescence measurements were performed on light-acclimated biofilms to assess the effective quantum yield of charge separation in photosystem II PSII, either under the incubator light conditions or during the measurement of electron transport rate versus irradiance (ETR/I) curves. Three slides were sampled in the middle of light period (8 h ca.) at the initial stage of biofilm development, immersed in culture medium to avoid desiccation, inhibition of photosynthesis, and/or damage to photosystems, and positioned on a specifically designed grid so that biofilm surface could be virtually divided into 27 spots (8 × 8 mm each). The fiberoptic was kept at 10 mm above biofilm surface using a holder to obtain a homogeneous field of illumination (Figure 1c). After PAM measurements, the slides were put back in the same position in the light chamber and then re-used for fluorescence assessment in the following samplings, i.e., at the active and mature stage of biofilm development.

The effective quantum yield of charge separation in PSII (ΔF/F_m_’) of the light-acclimated biofilms was calculated according to [49] the following:

ΔF/F_m_’ = (F_m_’−F)/F_m_’, (1)

where F is the steady-state fluorescence of the light-adapted sample and F_m_’ is the maximum light-adapted fluorescence after the application of a single saturating light pulse (800 ms, 3000 μmol photon m^-2^ s^-1^) [49,50]. ΔF/F_m_’ was measured on 27 spots of three slides (81 replicates) to take into account the heterogeneity of microalgal distribution. Thereafter, ETR/I curves were recorded on three spots on each slide providing nine replicates for each sampling. The position of the spots was chosen randomly at the initial sampling and then kept constant in the active and mature phase. ETR/I curves were measured by exposing the sample to eight increasing PFDs, up to 720 μmol photon m^-2^ s^-1^. Each period of actinic light lasted 10 s before the saturation pulse was applied to determine ΔF/F_m_’. The internal halogen lamp (20 W) of the Mini-PAM served as light source for saturation pulse and for continuous actinic illumination. The photosynthetic electron transport rate (ETR) between PSII and PSI was calculated from the estimate of ΔF/F_m_’ at different PFDs in actinic light according to the expression:

ETR = ΔF/F_m_’ · PFD · 0.5 · a*_PSII_, (2)

where 0.5 accounts for the excitation of both PSII and PSI and a*_PSII_ is the optical cross section of PSII. Since we could not measure a*_PSII_, relative ETR was calculated as ΔF/F_m_’ ∙ PFD ∙ 0.5.

The ETR/I curves were fitted by means of an automatic spreadsheet based on linear regression for estimating ETR per light intensity and a chi-square minimization for the exponential function ETR = ETR_max_ (1 -e ^−α I ETRmax^) [51]. From the fit of the maximum rate of relative ETR (rel.ETR_max_), the initial slope, i.e., photosynthetic efficiency (α) and the light saturation parameter (I_k_ = rel.ETR_max_/α) were calculated.

### 2.3. Biomass Estimation

Phototrophic biomass was assessed by determining chlorophyll *a* concentration per unit area (mg m^−2^). Chlorophyll *a* was extracted overnight in 90% acetone and then quantified spectrophotometrically [52].

Biomass productivity was determined as dry weight (DW g m^−2^ d^−1^) by oven drying samples at 60 °C for 72 h. Samples were then burnt at 500 °C for 1 h to determine organic biomass (phototrophs + heterotrophs) as ash-free dry weight (AFDW) per unit area (mg m^−2^) [53].

### 2.4. Data Elaboration

To test the influence of the experimental conditions and their interactions on all biofilm photosynthetic parameters, the standard ANOVA provided by the Past software (v 3.2.5) was used in combination with the Tukey Honestly Significant Difference (HSD) test for multiple post-hoc testing or Mann–Whitney test. The effect of these environmental conditions on initial and latest phases of biofilm development was evaluated by repeated ANOVA. Correlation between Chl *a* and AFDW was assessed by applying the Spearman’s rho-statistic [54].

## 3. Results and Discussion

### 3.1. Biofilm Growth

**Figure 2:**
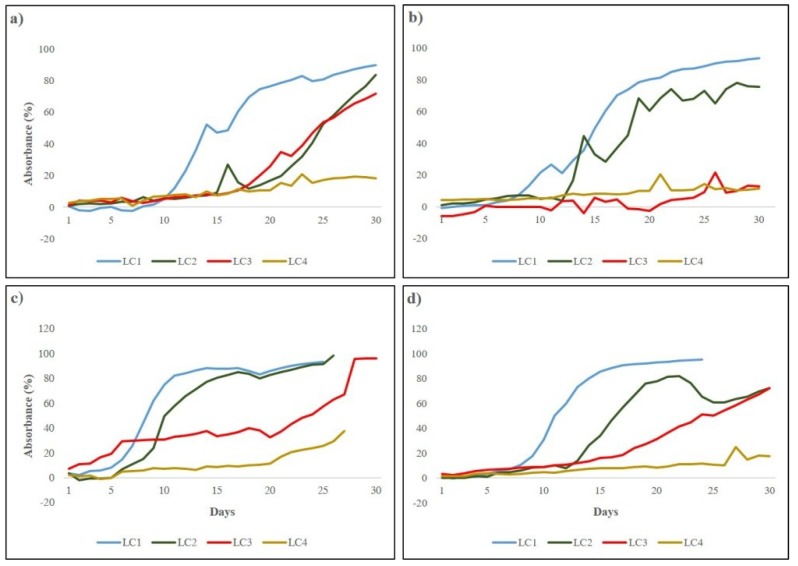
Growth curves of LC1, LC2, LC3, and LC4 biofilms in Run1 (**a**), Run2 (**b**), Run3 (**c**), Run4 (**d**) obtained by transforming the on-line recorded light transmitted values in light absorbance.

Biofilm development in the microcosm was monitored through an online and automatic system that continuously recorded the light transmitted through biofilm thickness over experimental time, allowing the calculation of absorbance and the subsequent construction of growth curves [40,55]. Growth of biofilms was witnessed by the increasing light absorbance (Figure 2). 

A sigmoidal trend was observed for biofilms grown at the higher irradiances (LC1 and LC2) in all runs, diversely from most of the cultures under lower irradiances (LC3 and LC4) that reached only the initial and active phase. Initial phase lasted between 7 (LC1 Run3) and 14 days (LC2 Run1) for high light cultures and between 18 (LC3 Run1, LC3 Run4) and 30 days (LC4 Run4) for the low light ones. Irradiance appeared to influence biofilm development, in particular the duration of the initial phase, i.e., the higher the irradiance, the shorter resulted the lag phase. This is more evident in LC1 cultures, likely due to the dominance of unicellular green algae which have higher growth rates [40]. The slope of the curves, reflecting the active phase of biofilm development, was more pronounced in LC1 and LC2 cultures irrespective of the different temperature and flow conditions tested, reflecting the fast growth of phototrophs. In LC3 and LC4, the active phase was not completed except for LC3 biofilms in Run3 (Figure 2c). Complete development of a mature community was obtained only in cultures at 120 µmol photons m^−2^ s^−1^ (LC1) in all runs when biofilms were dominated by filamentous cyanobacteria [40] able to produce high amounts of exopolysaccharides [38], allowing the establishment of complex and structured mature communities. The highest light intensity tested appeared to be the most favorable for biofilm formation and development as the lag phase was shorter, the active growth phase was fast, and mature communities were formed in all runs within the experimental time. 

**Figure 3:**
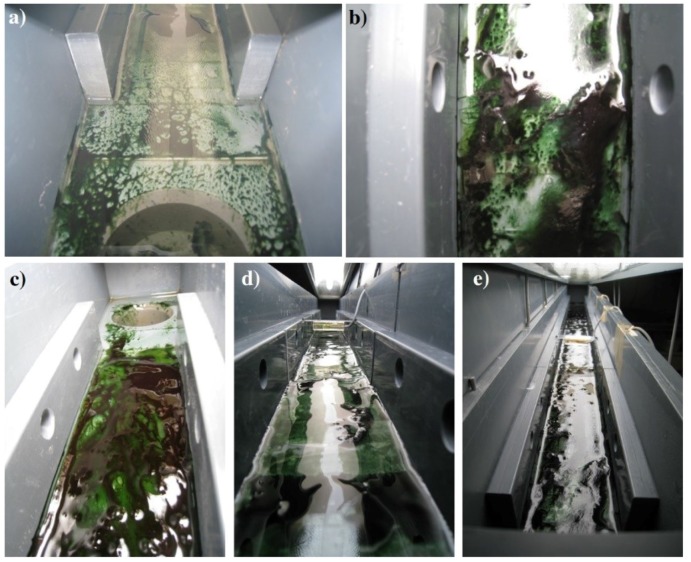
Details of LC1 biofilms growing in the incubator at the mature phase of development (**a**–**c**). Light sensor positioned under the lamp (**d**,**e**).

When observed at the macroscopic level, biofilm biomass accumulation on the slides was patchy at the initial phase of development reflecting the adhesion of the pioneer microorganisms during the colonization process [37]. The following active phase was characterized by an increasing and more homogeneous coverage of the substrata which ended to be completely colonized in the mature stage (Figure 3). 

Biofilm detachment from the slides occurred in a number of cases, visible from the concomitant appearance of bare areas on the slides and biofilm flocs in the circulating medium, these representing the dispersion, i.e., the last phase of biofilms development. Beyond self-detachment, which occurs when losses, due for example to cell death, grazing, pathogens, etc., become equal to growth, other factors (e.g., flow disturbance, temperature, light and nutrient availability) may have an impact [20,56]. Nutrient concentration does not represent a limitation, as long as N:P ratio is maintained, when biofilms are grown on wastewater in both indoor and outdoor photobioreactors. Therefore, optimization of flow regime, temperature and light intensity are the main parameters to focus on for high quality biomass production when the process is scaled up to real plant operations. Additionally, biomass harvesting frequency has to be assessed in order to ensure an effective treatment of WWs.

### 3.2. Photosynthetic Characteristics

**Figure 4:**
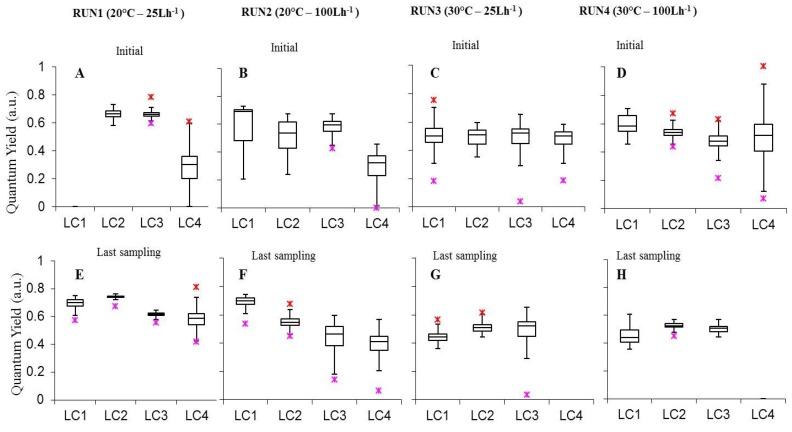
Box-plots relative to the comparison of quantum yield (ΔF/F_m_’) of photosystem II (PSII) measurements determined on all light-acclimated biofilms (Run1–Run4) at initial phase (**A**–**D**) and last sampling day (**E**–**H**). Run1-LC1in = not sampled.

**Figure 5:**
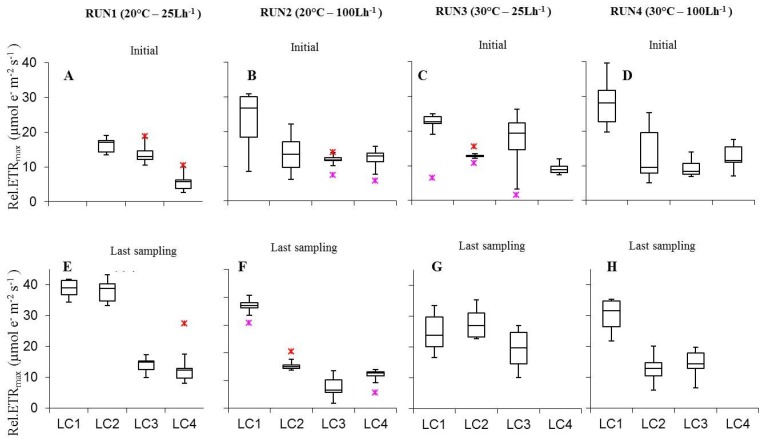
Box-plots relative to the comparison of rel.ETR_max_ values determined on all light-acclimated biofilms (Run1–Run4) at initial phase (**A**–**D**) and last sampling day (**E**–**H**). Run1-LC1in = not sampled.

**Figure 6:**
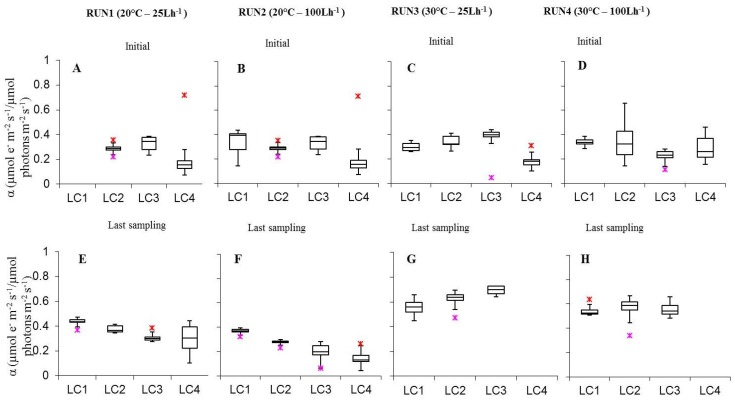
Box-plots relative to the comparison of α values determined on all light-acclimated biofilms (Run1–Run4) at initial phase (**A**–**D**) and last sampling day (**E**–**H**). Run1-LC1in = not sampled.

**Figure 7:**
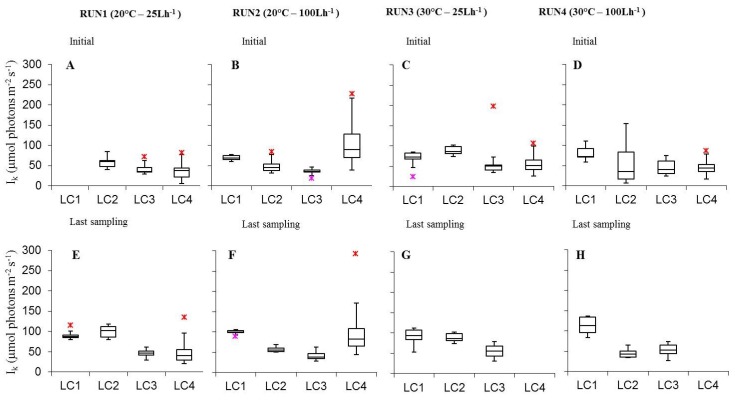
Box-plots relative to the comparison of I_k_ values determined on all light-acclimated biofilms (Run1–Run4) at initial phase (**A**–**D**) and last sampling day (**E**–**H**). Run1-LC1in = not sampled.

Results of photosynthetic performance of light-acclimated cultures, obtained after PAM measurements, are reported in Figure 4, Figure 5, Figure 6 and Figure 7 and in Tables S1–S8 are those obtained by statistical analysis to evaluate the effect of experimental conditions on photosynthetic parameters. 

The effective quantum yield of photosynthesis (ΔF/F_m_’) represents the efficiency of the whole-chain photosynthetic electron transport of steady-state photosynthesis in the light [57]. Values measured on the surface of intact biofilms, were included between 0.282 ± 0.015 (LC4in Run1) and 0.742 ± 0.001, with the latter occurring in 30-days biofilms at 60 µmol photons m^−2^ s^−1^, 20 °C and 25 L h^−1^ (LC2ls Run1). Most yield values were included between 0.5 and 0.7, falling in the ranges reported for natural biofilms from different environments, thus exposed at various ambient light intensities [31,58,59,60].

Analyzing separately the effect of experimental parameters on biofilm photosynthetic performance, we observed that, at initial stages, biofilm ΔF/F_m_’ was not affected by variations in light conditions (Figure 4A–D Table S1). Conversely, in the last experimental day in Run1 ΔF/F_m_’ increased from 15 to 60 μmol photon m^−2^ s^−1^ (LC3ls-LC4ls *p* = 0, LC2ls-LC3ls *p* = 0) followed by a significant decrease from 60 to 120 μmol photon m^−2^ s^−1^ (*p* = 0.00001254). In Run2 a significant increase occurred from 15 to 120 μmol photon m^−2^ s^−1^ (Run2 LC3ls-LC4ls *p* = 0.0001251, LC2ls-LC3ls *p* = 0, LC1ls-LC2ls *p* = 0) (Table S2). 

Moreover, in all Runs but Run2, cultures at 60 μmol photon m^−2^ s^−1^ showed significantly higher yield than biofilms grown at the highest irradiance (*p* < 0.05 see Table S2) indicating a proper functioning of the phototrophic community at this irradiance and prospecting a suitable light condition for cultivating phototrophic biofilms in photobioreactors for WWT.

The effect of temperature was clearer in biofilms at the highest light intensities were significantly higher values were obtained at 20 °C than 30 °C at both flow velocities (*p* < 0.05 see Table S2), likely because rates of enzymatic reactions involved in electron transport are temperature-dependent [61,62,63,64]. High temperature represents an excess energy and may lead to an imbalance between the energy supply and energy consumption by the redox reactions in the electron transport chain resulting in a progressive photoinactivation of PSII reaction centers [63]. 

As for the effect of flow rate on biofilm photosynthesis, initial stages yield showed a scattered distribution. The most relevant outcome was obtained at 60 μmol photon m^−2^ s^−1^ where LC2 cultures attained significantly higher yields at 25 L h^−1^ than 100 L h^−1^ at 20 °C (Run1 LC2in-Run2 LC2in *p* = 0). This was also recorded at the runs’ end at 20 °C (Run1 LC2ls-Run2 LC2ls *p* = 0) while no significant difference was found between yields at different flow rates at 30 °C (*p* > 0.05 Table S2). 

The combined effect of light, temperature, and flow was also evaluated on biofilms photosynthesis. In initial stage biofilms ΔF/F_m_’ was significantly higher at 30 μmol photon m^−2^ s^−1^, 20 °C, 25 L h^−1^ (Run1-LC3) than at all other conditions (*p* < 0.05 Table S1) suggesting that these irradiance, temperature, and flow values were the most favorable for light utilization in the photosynthetic process. Noteworthy, ΔF/F_m_’ was higher at 60 μmol photon m^−2^ s^−1^, 20 °C, 25 L h^−1^ (Run1-LC2) than at almost all the other conditions (Table S1). The only exception was the non-significant difference between Run1-LC2 and Run1-LC3 (*p* = 1). In the last phase, 60 μmol photon m^−2^ s^−1^, 20 °C and 25 L h^−1^ (Run1 LC2) were the conditions providing higher yield values (*p* < 0.05 Table S2). This suggested that to maintain biofilm photosynthesis in optimal conditions, and all the processes related to it, which are essential for WWT, a full-scale photobioreactor might be operated at 20 °C, 25 L h^−1^ and at an irradiance of 30 or 60 μmol photon m^−2^ s^−1^, initially, and then at 60 μmol photon m^−2^ s^−1^.

Photoacclimation of cultured biofilms to different experimental irradiances was indicated also by variations in ETR/I curve parameters such as rel.ETR_max_, I_k_, and α (Figure 5, Figure 6 and Figure 7). Rel.ETR_max_ (Figure 5) varied between 5.5 ± 0.9 and 43.4 ± 1.3 µmol e^−^ m^−2^ s^−1^ (LC4in Run1 and LC1ac Run2, respectively). 

Plotting rel.ETR_max_ variations with irradiance did not show coherent effects both in initial and last day biofilms (Figure 5). In both cases, the most relevant result was the significant increase from 60 to 120 μmol photon m^−2^ s^−1^ in Run2 (*p* = 0.0005674), reflecting the photoacclimation of the benthic phototrophic communities at increasing irradiance [32,60,65]. At higher temperature initial and last day biofilms did not show significant rel.ETR_max_ variations at both flow conditions (Tables S3 and S4); except for the higher rel.ETR_max_ of LC1ls cultures at 25 L h^−1^ at 20 °C (*p* = 0.0004123). As for the effect of flow rate, only few variations were significant both in samples at the initial phase and at the last run day (Tables S3 and S4). The interaction of light, temperature and flow rate variation on rel.ETR_max_ yielded different results between initial biofilms and cultures sampled at the last day. In the former cases, rel.ETR_max_ was significantly higher at 120 μmol photon m^−2^ s^−1^ and 100 L h^−1^, both at 20 °C and 30 °C, than at most of the parameters combinations tested (Table S3); while in the latter cases, rel.ETR_max,_ significantly increased at 60 and 120 μmol photon m^−2^ s^−1^, at 20 °C and 25 L h^−1^ (Table S4).

As for photosynthetic efficiency (α), it was included between 0.14 ± 0.02 (LC4 Run2) and 0.43 ± 0.01 (LC1ac Run1). The visual analysis of α values in relation to irradiance showed that in initial biofilms of Run1–3, α increased from 15 to 30 μmol photon m^−2^ s^−1^ and decreased from 30 to 60 μmol photon m^−2^ s^−1^ (Figure 6). Nevertheless, only the variation between LC3 and LC4 at 20 °C and 100 L h^−1^ was significant (*p* = 0.004918) (Table S5). In biofilms collected at runs’ end, α increased at 20 °C, both at low and high flow rate, from 15 to 120 μmol photon m^−2^ s^−1^. Some of the variations observed were significant, i.e., Run2LC1-Run2LC2 (*p* = 0.01241), Run2LC2-Run2LC3 (*p* = 0.03837) (Table S6). At the initial stage of development, temperature variations did not have a significant effect on α while at the last sampling day some significant variations occurred. In detail, at low flow, α was higher at 20 °C than at 30 °C (*p* < 0.05 see Table S6) in biofilms at both the highest light intensities while, at high flow, only at 120 μmol photon m^−2^ s^−1^ (*p* = 0.006375). Variation of α with temperature, likewise ΔF/F_m_’, depends on the photochemical reactions that are temperature dependent (e.g enzymes of photophosporylation and electron transport and plastoquinone diffusion). As for the flow rate variation, similarly to temperature, no significant differences were observed in initial biofilms, at both temperatures. In cultures at the runs’ end at 20 °C, significant higher α values were obtained at low than high flow at LC2 (*p* = 0.002964), LC3 (*p* = 0.0001108), LC4 (*p* =1.894E-8). Regarding the interaction between light intensity, temperature, and flow rate, for biofilms in the initial phase it was not possible to find the conditions optimizing α, while for cultures sampled at the runs’ end two combinations were identified, 60 and 120 μmol photon m^−2^ s^−1^, 20 °C, 25 L h^−1^ (Table S6).

I_k_ ranged (Figure 7) between 34.8 ± 3.2 (LC3in Run2) and 128.1 ± 5.2 μmol photon m^−2^ s^−1^ (LC1ac Run2). Most values of rel.ETR_max_ and I_k_ obtained matched those found for sub-tidal phototrophic biofilms from different geographical areas [32,60]. In biofilms at initial stage and at the last experimental day, light variations influenced I_k_ only in few cases (Tables S7 and S8) while temperature had no effect on cultures at both phases of development, except for initial biofilms at 15 μmol photon m^−2^ s^−1^ and 100 L h^−1^ with higher values at 20 °C than at 30 °C (*p* = 0.005464). As for the flow rate, I_k_ did not have a significant variation in initial cultures while being significantly higher at 20 °C and lower at 30 °C at 100 L h^−1^ at almost all light conditions (Table S8). For the combined effect of light, temperature, and flow rate results were different between initial biofilms and cultures at the last sampling day. In the former cases, I_k_ was significantly higher at the lowest light intensity, low temperature and high flow (Table S7); in the latter cases, I_k_ was significantly higher at 60 and 120 °C μmol photon m^−2^ s^−1^, 20 °C, 25 L h^−1^ (Table S8).

**Figure 8:**
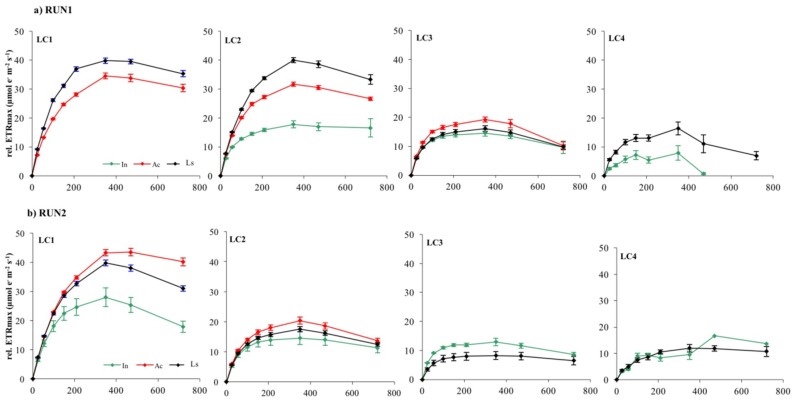
Electron Transport Rates *versus* Irradiance relative to LC1, LC2, LC3, and LC4 biofilms at different stage of development in Run1 (**a**) and Run2 (**b**). Each curve represents the mean of nine replicates.

**Figure 9:**
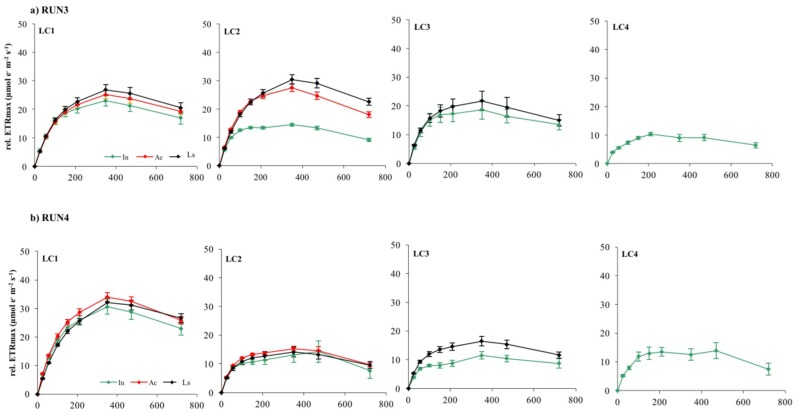
Electron transport rates versus irradiance relative to LC1, LC2, LC3, and LC4 biofilms at different stage of development in Run3 (**a**) and Run4 (**b**). Each curve represents the mean of nine replicates.

Photoinhibition, measured as the reduction of rel.ETR during ETR/I curve registration, occurred in almost all LC1, LC2, and LC3 samples at 470 μmol photon m^−2^ s^−1^, irrespective of growth irradiance and biofilm developmental stage (Figure 8 and Figure 9).

LC4 cultures showed photoinhibition at lower but, nevertheless, quite moderate irradiance (210–350 mmol photon m^−2^ s^−1^) considering that they were grown at the lowest light. This result suggests that the phototrophic community studied had the potential to acclimate to higher irradiances than those tested in the incubator cultures. Nevertheless, occurrence of photoinhibition in benthic communities during laboratory measurements and its relationship with the growth light regime is still quite controversial [33]. Photoinhibition may be often hidden by the self-shading effect within the biofilm matrix [28]. If the matrix is deep enough, decreases in the rate of oxygen production in the uppermost layer of biofilms with high irradiance are compensated for by increased photosynthesis of subsurface phototrophs, which receive only saturating or subsaturating irradiance [28,32,66]. On the contrary, fluorescence measurement of photosynthesis is derived mainly from the uppermost layers of phototrophs due to light attenuation with depth, particularly in thick mature biofilms [33,35,67]. Thus, the occurrence of photoinhibition reflects more the acclimation of surface cells to incident light rather than self-shading effects within the biofilm [33]. Therefore, ETR/I curves of LC1 and LC2 biofilms probably reflected the photoinhibition of phototrophs in the uppermost layers, particularly in the active and mature phases of development when communities were greatly stratified.

### 3.3. Biomass Production

Biomass accumulation has previously been shown to be linearly related to the decrease of subsurface light below the substratum [40,48]. The increase of biomass measurements over time indicated that natural mixed inocula were able to grow in culture after biofilm structure alteration caused by initial homogenizing treatment. 

**Figure 10:**
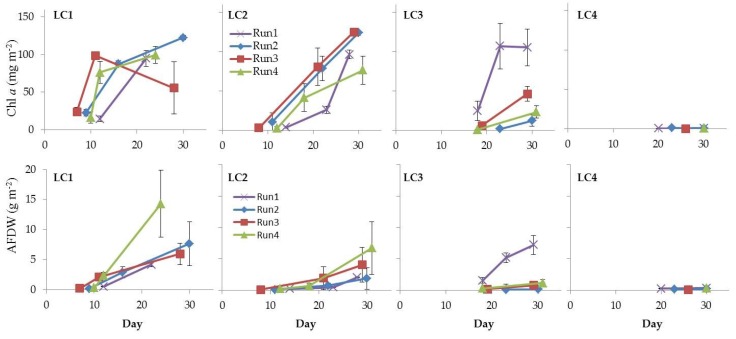
Temporal development of Chl *a* and AFDW in LC1, LC2, LC3, and LC4 biofilms (Run1–Run4).

Chl *a* concentration, used to estimate the phototrophic biomass in the cultured biofilms, ranged between 0.16 and 125.10 ± 1.52 mg m^−2^ (LC4in Run1 and LC2ls Run1, respectively, Figure 10). Mean values were in the range reported for 7-days biofilms grown in situ on artificial substrata (up to 252.6 mg m^−2^) [46] and for freshwater biofilms of rivers and streams impacted by nutrient-rich urban and agricultural waste discharges [68,69,70]. 

Looking at Chl *a* trend, at 120 and 60 µmol photons m^−2^ s^−1^, Chl *a* concentration increased over time following biofilm development, up to approximately 120 mg m^−2^ in final cultures. This suggested that 60 µmol photons m^−2^ s^−1^ was sufficient for biofilm development and higher intensity did not appear to exert any additional effect. Nevertheless, at this irradiance a longer lag phase was required for biofilm growth than at 120 µmol photons m^−2^ s^−1^. This might have implications in the selection of the light condition for culturing microbial-algal consortia in a wastewater photobioreactor. 

Whole biofilm biomass, estimated as AFDW, grew up to 14.30 ± 3.20 g m^−2^ (LC1ls Run4) (Figure 10). This value fell in the range reported for natural biofilms from streams both subjected or not to nutrient pollution [71,72]. Lower values, between ca. 23 and 37 mg m^−2^, were achieved in biofilms grown in artificial streams with wastewater at different sewage percentage [70]. A significant positive correlation was found between Chl *a* and AFDW (Table S9), suggesting that the phototrophic community represented the main component of the whole biofilm. This was confirmed by the autotrophic index (AI), which is a means of determining the metabolism/trophic nature of biofilms [53]. Most values were below 50 (Table 1), indicating that biofilms were autotrophs-dominated [73]. 

Values of ca. 140 were obtained in LC1 mature cultures at the highest light in Run3 and Run4, indicating a balance between the photo- and heterotrophic components [71,74]. Nevertheless, in LC1 cultures in Run3 and Run4 the heterotrophic component became, in terms of biomass, more important as biofilms grew. The AI values, in fact, increased over time due to the increase of AFDW and the saturation of Chl *a* in mature communities. The raise of heterotrophs might be explained by the increased amount, in mature biofilms at high light intensity, of algal exudates that represent a high-quality organic substrate for bacteria [71,75,76]. Wagner et al. [77] observed a shift towards bacterial consumption of DOC of autochthonous origin in phototrophic stream biofilms at high light intensity. As explained, cultured mature biofilms were dominated by filamentous cyanobacteria able to produce high amounts of exopolysaccharides [38,40]. Finally, heterotrophs strongly dominated cultures at the lowest irradiances [37] where values were above 200 in all samples.

Biomass productivity was included between 0.01 (LC3in Run2, LC4in in Run2 and Run3) and 2.10 ± 0.36 (LC1ma Run4) g DW m^−2^ d^−1^ (Table 2). 

Productivity was consistent with those reported [78] for mixed biofilms composed of green algae and diatoms in a lab-scale biofilm reactor (0.97–2.08 g DW m^−2^ d^−1^). The highest productivity values obtained here were higher than those attained with three different biofilm-forming cyanobacterial isolates grown in the same system [15] and with the green alga *Chlorella vulgaris* grown in a rotating algal bioreactor [17]. Higher values were reported for an algal biofilm grown in a photobioreactor [26]. 

## 4. Conclusions

This study provided new insights into photosynthetic activity of light-acclimated cultures, contributing to the knowledge of wastewater biofilm ecophysiology, in light of their exploitation in water bioremediation. The difficulty of studying these communities in nature, due to the complex interactions among several environmental factors, was overridden in the flow lane system, which allowed the investigation of key variables that influence photosynthetic performance.

- The tested light–dark cycle appeared to be suitable for community development and functioning.
- It was possible to identify light intensity, temperature, and flow value favoring photosynthetic activity, i.e., 60 µmol photons m^−2^ s^−1^, 20 °C, 25 l^−1^. This has implications for biomass accumulation and nutrient uptake related to photosynthesis. Therefore, this set of values appears to be appropriate for the optimal operating of a WWT photobioreactor that purifies municipal sewage by means of mixed phototrophic biofilms.
- Further development would be in the cultivation of these microbial communities in wastewater effluents to evaluate the treatment efficiency at the set of conditions here identified.

## Figures and Tables

**Table 1:** Mean values ± standard error of AI at initial (in), active (ac), 85% light absorption, last sampling day of biofilm development in the four runs performed. ns = not sampled.

Light Chamber	Sample	*Run1*	*Run2*	*Run3*	*Run4*
LC1	in	ns	9 ± 5	11 ± 2	24 ± 7
	ac	38 ± 5	33 ± 6	−	31 ± 4
	85%	44 ± 1	−	21 ± 2	−
	ma	ns	63 ± 18	144 ± 57	141 ± 23
LC2	in	62 ± 11	5	45 ± 22	131 ± 73
	ac	19 ± 2	9 ± 3	23 ± 11	23 ± 12
	ld	21 ± 1	15 ± 8	33 ± 13	87 ± 30
LC3	in	94 ± 52	55 ± 10	35 ± 15	331 ± 114
	ac	57 ± 20	−	−	−
	ld	68 ± 1	15 ± 7	18 ± 5	48 ± 7
LC4	in	860 ± 165	454 ±367	363	742
	ld	655 ± 284	748 ± 382	−	−

**Table 2:** Mean values ± standard error of biomass productivity (DW g m^−2^ d^−1^) at initial (in) and active (ac) biofilms and cultures at last sampling day (ls) in the four runs performed. ns = not sampled.

Light Chamber	Sample	*Run1*	*Run2*	*Run3*	*Run4*
LC1	in	ns	0.14 ± 0.04	0.26 ± 0.04	0.20 ± 0.02
	ac	0.25 ± 001	0.80 ± 0.08	1.09 ± 0.15	0.91 ± 0.04
	ls	0.81 ± 0.08	1.08 ± 0.09	1.36 ± 0.40	2.10 ± 0.36
LC2	in	0.04 ± 0.00	0.32 ± 0.18	0.04 ± 0.00	0.06 ± 0.02
	ac	0.13 ± 0.01	0.20 ± 0.04	0.37 ± 0.10	0.24 ± 0.06
	ls	0.37 ± 0.01	0.33 ± 0.12	0.51 ± 0.09	0.70 ± 0.21
LC3	in	0.14 ± 0.02	0.01 ± 0.00	0.03 ± 0.01	0.04 ± 0.01
	ac	0.29 ± 0.11	−	−	−
	ls	0.51 ± 0.05	0.09 ± 0.06	0.08 ± 0.01	0.21 ± 0.05
LC4	in	0.03 ± 0.00	0.01 ± 0.01	0.01 ± 0.00	0.03 ± 0.01
	ls	0.02 ± 0.00	0.01 ± 0.01	−	−

## Supplementary Materials

The following are available online at https://www.mdpi.com/2076-2607/7/8/252/s1, Table S1: Results of ANOVA and Tukey’s HSD testing with factors irradiance, temperature and flow on ΔF/Fm’ in initial biofilms. Tukey’s Q below the diagonal, *p* value above the diagonal. Significant comparisons are red. Table S2: Results of ANOVA and Tukey’s HSD testing with factors irradiance, temperature and flow on ΔF/Fm’ in biofilms at the last sampling day. Tukey’s Q below the diagonal, *p* value above the diagonal. Significant comparisons are red. Table S3: Results of ANOVA and Tukey’s HSD testing with factors irradiance, temperature and flow on rel.ETRmax in initial biofilms. Tukey’s Q below the diagonal, *p* value above the diagonal. Significant comparisons are red. Table S4: Results of ANOVA and Mann–Whitney test testing with factors irradiance, temperature and flow on rel.ETRmax in biofilms at the last sampling day. Significant comparisons are red. Table S5: Results of ANOVA and Tukey’s HSD testing with factors irradiance, temperature and flow on α in initial biofilms. Tukey’s Q below the diagonal, *p* value above the diagonal. Significant comparisons are red. Table S6: Results of ANOVA and Tukey’s HSD testing with factors irradiance, temperature and flow on α in biofilms at the last sampling day. Tukey’s Q below the diagonal, *p* value above the diagonal. Significant comparisons are red. Table S7: Results of ANOVA and Tukey’s HSD testing with factors irradiance, temperature and flow on Ik in initial biofilms. Tukey’s Q below the diagonal, *p* value above the diagonal. Significant comparisons are red. Table S8: Results of ANOVA and Tukey’s HSD testing with factors irradiance, temperature and flow on Ik in biofilms at the last sampling day. Tukey’s Q below the diagonal, *p* value above the diagonal. Significant comparisons are red. Table S9: Results of Spearman’s rho-statistic applied to test the correlation between Chl a and AFDW.
