# Supplementary material for: Wastewater Biofilm Photosynthesis in Photobioreactors
Source: Microorganisms. 2019 Aug 10;7(8):252. doi: 10.3390/microorganisms7080252 (PMC6723877; doi:10.3390/microorganisms7080252)
Supplement: Supplementary file 1 [file microorganisms-07-00252-s001.pdf]

microorganisms

MDPI

Table 1 Supplementary Information. Results of ANOVA and Tukey's HSD testing with factors irradiance, temperature and flow on ΔF/Fm' in initial biofilms. Tukey's Q below the diagonal, p value above the diagonal. Significant comparisons are red.

		**Run1** (20°C, 25 l h^{-1})			**Run2** (20°C, 100 l h^{-1})				**Run3** (30°C, 25 l h^{-1})				**Run4** (30°C, 100 l h^{-1})			
		LC2	LC3	LC4	LC1	LC2	LC3	LC4	LC1	LC2	LC3	LC4	LC1	LC2	LC3	LC4
**Run1** (20°C, 25 l h^{-1})	LC2		1	0	0.00045	0	4.47E-05	0	0	0	0	0	0.001338	5.26E-13	0	0
	LC3	0.09083		0	0.000334	0	3.22E-05	0	0	0	0	0	0.00101	2.89E-13	0	0
	LC4	34.33	34.42		0	0	0	0.9972	0	0	0	0	0	0	0	0
**Run2** (20°C, 100 l h^{-1})	LC1	6.51	6.601	27.84		0.000122	1	0	1.77E-08	1.70E-07	3.07E-10	3.55E-11	1	0.06936	5.91E-10	1.81E-06
	LC2	13.41	13.5	20.96	6.899		0.001109	0	0.9657	0.9973	0.6822	0.4546	3.59E-05	0.9614	0.7379	1
	LC3	7.183	7.273	27.17	0.6724	6.227		0	3.30E-07	2.71E-06	7.41E-09	9.80E-10	1	0.2395	1.36E-08	2.41E-05
	LC4	32.84	32.93	1.703	26.31	19.39	25.63		0	0	0	0	0	0	0	0
**Run3** (30°C, 25 l h^{-1})	LC1	15.62	15.71	18.76	9.112	2.213	8.44	17.17		1	1	0.9999	3.66E-09	0.1036	1	1
	LC2	15.11	15.2	19.27	8.596	1.697	7.924	17.68	0.5162		0.9997	0.9947	3.81E-08	0.2552	0.9999	1
	LC3	16.49	16.58	17.9	9.976	3.077	9.304	16.3	0.864	1.38		1	5.54E-11	0.01454	1	0.9889
	LC4	16.92	17.01	17.47	10.41	3.509	9.736	15.87	1.296	1.812	0.432		5.85E-12	0.004522	1	0.9374
**Run4** (30°C, 100 l h^{-1})	LC1	6.166	6.257	28.18	0.3445	7.243	1.017	26.65	9.457	8.941	10.32	10.75		0.03215	1.10E-10	4.44E-07
	LC2	11.16	11.26	23.2	4.655	2.244	3.982	21.64	4.458	3.941	5.322	5.754	4.999		0.01972	0.5282
	LC3	16.33	16.42	17.94	9.841	2.964	9.171	16.35	0.7573	1.272	0.1041	0.5347	10.18	5.201		0.9937
	LC4	14.53	14.63	19.84	8.024	1.125	7.352	18.26	1.088	0.5716	1.952	2.384	8.369	3.37	1.842	

Table 2 Supplementary Information. Results of ANOVA and Tukey's HSD testing with factors irradiance, temperature and flow on ΔF/Fm' in biofilms at the last sampling day. Tukey's Q below the diagonal, p value above the diagonal. Significant comparisons are red.

		**Run1** (20°C, 25 l h^{-1})				**Run2** (20°C, 100 l h^{-1})				**Run3** (30°C, 25 l h^{-1})			**Run4** (30°C, 100 l h^{-1})		
		LC1	LC2	LC3	LC4	LC1	LC2	LC3	LC4	LC1	LC2	LC3	LC1	LC2	LC3
**Run1** (20°C, 25 l h^{-1})	LC1		1.25E-05	0	0	1	0	0	0	0	0	0	0	0	0
	LC2	7.493		0	0	1.59E-07	0	0	0	0	0	0	0	0	0
	LC3	12.33	19.82		0.006737	3.26E-13	5.41E-10	0	0	0	0	0	0	0	0
	LC4	17.89	25.39	5.564		0	0.1321	0	0	0	6.46E-11	0	0	1.63E-06	1.08E-13
**Run2** (20°C, 100 l h^{-1})	LC1	1.091	8.584	11.24	16.8		0	0	0	0	0	0	0	0	0
	LC2	22.17	29.66	9.837	4.273	21.07		0	0	0	0.001956	1.46E-12	0	0.3105	4.96E-05
	LC3	38.01	45.45	25.75	20.23	36.92	15.98		0.000125	1	2.23E-10	0.02472	1	0	3.92E-08
	LC4	45.14	52.63	32.81	27.25	44.05	22.98	6.853		7.24E-05	0	0	4.99E-06	0	0
**Run3** (30°C, 25 l h^{-1})	LC1	38.13	45.62	25.8	20.24	37.04	15.97	0.1133	7.01		2.86E-10	0.03006	1	0	5.05E-08
	LC2	28.16	35.66	15.83	10.27	27.07	5.997	10.02	16.98	9.969		0.02899	8.68E-09	0.9483	0.9999
	LC3	33.15	40.65	20.83	15.26	32.06	10.99	5.059	11.99	4.977	4.992		0.1373	3.15E-05	0.2585
**Run4** (30°C, 100 l h^{-1})	LC1	37.41	44.9	25.08	19.51	36.32	15.24	0.8336	7.735	0.7249	9.244	4.252		2.84E-14	1.07E-06
	LC2	25.91	33.4	13.58	8.019	24.82	3.746	12.26	19.23	12.22	2.251	7.243	11.49		0.4931
	LC3	29.28	36.78	16.95	11.39	28.19	7.117	8.907	15.86	8.849	1.12	3.872	8.124	3.371	

Table 3 Supplementary Information. Results of ANOVA and Tukey’s HSD testing with factors irradiance, temperature and flow on rel.ETR$_{max}$ in initial biofilms. Tukey's Q below the diagonal, p value above the diagonal. Significant comparisons are red.

		**Run1** (20°C, 25 l h^{-1})			**Run2** (20°C, 100 l h^{-1})				**Run3** (30°C, 25 l h^{-1})				**Run4** (30°C, 100 l h^{-1})			
		LC2	LC3	LC4	LC1	LC2	LC3	LC4	LC1	LC2	LC3	LC4	LC1	LC2	LC3	LC4
**Run1** (20°C, 25 l h^{-1})	LC2		0.9971	0.001696	0.03957	9.97E-01	0.7089	0.918	0.787	0.9707	1	0.1158	1.59E-04	0.9523	0.2328	0.9203
	LC3	1.684		0.06908	0.00059	1	0.9998	1	0.1034	1	0.9699	0.8125	7.63E-07	1	0.9144	1
	LC4	6.317	4.742		3.66E-10	0.07085	0.4703	3.97E-01	4.71E-07	1.53E-01	4.98E-04	0.9719	2.70E-13	1.87E-01	9.71E-01	0.2357
**Run2** (20°C, 100 l h^{-1})	LC1	5.016	6.699	11.01		0.000567	1.05E-05	0.000163	0.9682	0.000153	0.1008	8.41E-08	0.9723	0.000103	1.07E-06	6.35E-05
	LC2	1.697	0.01391	4.729	6.713		0.9998	1.00E+00	1.01E-01	1.00E+00	9.68E-01	0.818	7.28E-07	1.00E+00	9.18E-01	1
	LC3	3.019	1.336	3.493	8.035	1.322		1	0.005511	1	0.4705	0.9994	7.49E-09	1	0.9999	1
	LC4	2.451	0.8766	3.644	7.143	0.8636	0.3728		0.03215	1	0.7662	9.96E-01	2.71E-07	1	0.9992	1
**Run3** (30°C, 25 l h^{-1})	LC1	2.845	4.529	8.978	2.17	4.543	5.864	5.113		0.04135	0.9356	9.89E-05	0.1528	0.03118	0.000585	0.02188
	LC2	2.15	0.4661	4.306	7.165	0.4522	0.8696	0.4406	4.995		0.8699	9.46E-01	1.57E-07	1	0.9822	1
	LC3	0.4731	2.157	6.759	4.543	2.17	3.492	2.894	2.372	2.623		4.64E-02	6.26E-04	0.8233	0.1122	0.757
	LC4	4.466	2.783	2.139	9.482	2.769	1.447	1.726	7.311	2.317	4.939		3.90E-11	9.67E-01	1.00E+00	0.9826
**Run4** (30°C, 100 l h^{-1})	LC1	7.151	8.835	13.01	2.136	8.849	10.17	9.141	4.306	9.301	6.678	11.62		9.95E-08	1.00E-09	5.71E-08
	LC2	2.282	0.5983	4.182	7.298	0.5844	0.7374	0.317	5.127	0.1322	2.755	2.184	9.433		0.99	1
	LC3	4.041	2.466	2.146	8.733	2.453	1.217	1.499	6.703	2.03	4.484	0.1367	10.73	1.907		0.9955
	LC4	2.442	0.7583	4.033	7.458	0.7444	0.5774	0.1673	5.287	0.2922	2.915	2.024	9.593	0.16	1.757	

Table 4 Supplementary Information. Results of ANOVA and Mann–Whitney test testing with factors irradiance, temperature and flow on rel.ETRmax in biofilms at the last sampling day. Significant comparisons are red.

		**Run1** (20°C, 25 l h^{-1})				**Run2** (20°C, 100 l h^{-1})				**Run3** (30°C, 25 l h^{-1})			**Run4** (30°C, 100 l h^{-1})		
		LC1	LC2	LC3	LC4	LC1	LC2	LC3	LC4	LC1	LC2	LC3	LC1	LC2	LC3
**Run1** (20°C, 25 l h^{-1})	LC1		0.4527	0.000412	0.00028	0.1577	0.000412	0.000412	0.000412	0.000412	0.00057	0.00179	0.001086	0.00041	0.000412
	LC2	0.4527		0.000412	0.00028	0.5365	0.000412	0.000412	0.000412	0.000574	0.00147	0.00179	0.01036	0.00041	0.000412
	LC3	0.000412	0.000412		0.09417	0.000412	0.5361	0.003569	0.0934	0.000574	0.00041	0.3165	0.000412	0.1992	0.6588
	LC4	0.00028	0.00028	0.09417		0.00028	0.00425	0.04994	0.8702	0.001669	0.001253	0.05768	0.000703	0.5954	0.153
**Run2** (20°C, 100 l h^{-1})	LC1	0.1577	0.5365	0.000412	0.00028		0.000412	0.000412	0.000412	0.000923	0.00147	0.00179	0.006193	0.00041	0.000412
	LC2	0.000412	0.000412	0.5361	0.00425	0.000412		0.000484	0.000792	0.001478	0.00041	0.3453	0.000412	0.07724	0.6588
	LC3	0.000412	0.000412	0.003569	0.04994	0.000412	0.000484		0.0575	0.000412	0.00041	0.005614	0.000412	0.04215	0.005387
	LC4	0.000412	0.000412	0.0934	0.8702	0.000412	0.000792	0.0575		0.000412	0.00041	0.02924	0.000412	0.4797	0.07012
**Run3** (30°C, 25 l h^{-1})	LC1	0.000412	0.000574	0.000574	0.001669	0.000923	0.001478	0.000412	0.000412		0.2161	0.2884	0.04226	0.000917	0.001086
	LC2	0.00057	0.00147	0.00041	0.001253	0.00147	0.00041	0.00041	0.00041	0.2161		0.0675	0.3311	0.000407	0.00041
	LC3	0.00179	0.00179	0.3165	0.05768	0.00179	0.3453	0.005614	0.02924	0.2884	0.0675		0.02156	0.0872	0.2629
**Run4** (30°C, 100 l h^{-1})	LC1	0.001086	0.01036	0.000412	0.000703	0.006193	0.000412	0.000412	0.000412	0.04226	0.3311	0.02156		0.00041	0.000412
	LC2	0.00041	0.00041	0.1992	0.5954	0.00041	0.07724	0.04215	0.4797	0.000917	0.000407	0.0872	0.00041		0.3533
	LC3	0.000412	0.000412	0.6588	0.153	0.000412	0.6588	0.005387	0.07012	0.001086	0.00041	0.2629	0.000412	0.3533	

Table 5 Supplementary Information. Results of ANOVA and Tukey's HSD testing with factors irradiance, temperature and flow on α in initial biofilms. Tukey's Q below the diagonal, p value above the diagonal. Significant comparisons are red.

		**Run1** (20°C, 25 l h^{-1})			**Run2** (20°C, 100 l h^{-1})				**Run3** (30°C, 25 l h^{-1})				**Run4** (30°C, 100 l h^{-1})			
		LC2	LC3	LC4	LC1	LC2	LC3	LC4	LC1	LC2	LC3	LC4	LC1	LC2	LC3	LC4
**Run1** (20°C, 25 l h^{-1})	LC2		0.9993	0.9978	0.9868	1	0.9934	0.1811	1	0.9962	0.9993	0.7239	0.997	0.9988	0.9969	1
	LC3	1.466		0.7127	1	0.9984	1	0.01122	1	1	1	0.1184	1	1	0.6871	1
	LC4	1.64	3.011		0.4781	0.999	0.5446	0.9261	0.9742	0.5921	0.7127	1	0.6101	0.6757	1	0.9844
**Run2** (20°C, 100 l h^{-1})	LC1	1.964	0.4979	3.477		0.9776	1	0.003518	0.9994	1	1	0.04526	1	1	0.4515	0.9985
	LC2	0.1194	1.586	1.528	2.084		0.988	0.2157	1	0.9927	0.9984	0.7774	0.994	0.9973	0.9985	1
	LC3	1.824	0.3581	3.346	0.1398	1.944		0.004918	0.9998	1	1	0.06018	1	1	0.5174	0.9995
	LC4	4.203	5.575	2.417	6.04	4.091	5.91		0.07768	0.006206	0.01122	0.9996	0.006772	0.009333	0.9368	0.09703
**Run3** (30°C, 25 l h^{-1})	LC1	0.5115	0.9548	2.118	1.453	0.6309	1.313	4.681		0.9999	1	0.4672	0.9999	1	0.9681	1
	LC2	1.726	0.2592	3.254	0.2387	1.845	0.09889	5.817	1.214		1	0.07311	1	1	0.5648	0.9998
	LC3	1.466	0	3.011	0.4979	1.586	0.3581	5.575	0.9548	0.2592		0.1184	1	1	0.6871	1
	LC4	2.987	4.454	1.155	4.952	2.868	4.812	1.409	3.499	4.713	4.454		0.07859	0.1021	1	0.5311
**Run4** (30°C, 100 l h^{-1})	LC1	1.688	0.2217	3.219	0.2762	1.807	0.1364	5.782	1.177	0.03751	0.2217	4.675		1	0.5828	0.9998
	LC2	1.548	0.08184	3.088	0.416	1.668	0.2762	5.651	1.037	0.1773	0.08184	4.536	0.1398		0.6492	1
	LC3	1.693	3.065	0.05027	3.53	1.581	3.4	2.366	2.171	3.307	3.065	1.101	3.272	3.141		0.9803
	LC4	0.3853	1.081	2	1.579	0.5047	1.439	4.563	0.1262	1.34	1.081	3.373	1.303	1.163	2.053	

Table 6 Supplementary Information. Results of ANOVA and Tukey's HSD testing with factors irradiance, temperature and flow on α in biofilms at the last sampling day. Tukey's Q below the diagonal, p value above the diagonal. Significant comparisons are red.

		**Run1** (20°C, 25 l h^{-1})				**Run2** (20°C, 100 l h^{-1})				**Run3** (30°C, 25 l h^{-1})			**Run4** (30°C, 100 l h^{-1})		
		LC1	LC2	LC3	LC4	LC1	LC2	LC3	LC4	LC1	LC2	LC3	LC1	LC2	LC3
**Run1** (20°C, 25 l h^{-1})	LC1		0.5103	0.000146	1.11E-05	0.252	1.03E-07	5.47E-14	0	2.46E-07	6.51E-05	0.08684	3.99E-08	7.69E-08	1.41E-07
	LC2	3.348		0.3	0.08843	1	0.002964	3.31E-10	5.92E-14	0.005687	0.2031	0.9976	0.001429	0.002372	0.003756
	LC3	7.149	3.802		1	0.5738	0.9426	0.000111	4.81E-09	0.9777	1	0.9902	0.8736	0.925	0.958
	LC4	7.993	4.558	0.6579		0.2385	0.9959	0.000434	1.89E-08	0.9993	1	0.8997	0.9827	0.9933	0.9977
**Run2** (20°C, 100 l h^{-1})	LC1	3.927	0.5788	3.223	3.964		0.01241	2.63E-09	1.01E-13	0.02233	0.4385	1	0.006375	0.01013	0.01538
	LC2	9.412	6.064	2.263	1.663	5.485		0.03837	9.81E-06	1	0.9789	2.81E-01	1	1	1
	LC3	14.39	11.04	7.241	6.772	10.46	4.979		0.6598	0.022	2.44E-04	4.11E-06	0.06716	0.04584	3.16E-02
	LC4	17.44	14.09	10.29	9.903	13.52	8.031	3.052		4.33E-06	1.24E-08	3.43E-10	2.33E-05	1.29E-05	7.33E-06
**Run3** (30°C, 25 l h^{-1})	LC1	9.155	5.808	2.006	1.4	5.229	0.2565	5.235	8.287		9.94E-01	0.378	1	1	1
	LC2	7.419	4.071	0.2697	0.3813	3.492	1.993	6.972	10.02	1.736		0.9714	9.41E-01	0.9702	0.986
	LC3	4.568	1.574	1.827	2.45	1.056	3.85	8.304	11.03	3.621	2.068		0.195	2.52E-01	0.3138
**Run4** (30°C, 100 l h^{-1})	LC1	9.688	6.34	2.539	1.947	5.762	0.2762	4.703	7.755	0.5328	2.269	4.097		1	1
	LC2	9.497	6.15	2.348	1.751	5.571	0.0855	4.893	7.945	0.342	2.078	3.927	0.1907		1
	LC3	9.32	5.972	2.17	1.569	5.393	0.09208	5.071	8.123	0.1644	1.901	3.768	0.3683	0.1776	

Table 7 Supplementary Information. Results of ANOVA and Tukey's HSD testing with factors irradiance, temperature and flow on I_k in initial biofilms. Tukey's Q below the diagonal, p value above the diagonal. Significant comparisons are red.

		**Run1** (20°C, 25 l h^{-1})			**Run2** (20°C, 100 l h^{-1})				**Run3** (30°C, 25 l h^{-1})				**Run4** (30°C, 100 l h^{-1})			
		LC2	LC3	LC4	LC1	LC2	LC3	LC4	LC1	LC2	LC3	LC4	LC1	LC2	LC3	LC4
**Run1** (20°C, 25 l h^{-1})	LC2		0.9968	0.9675	1	1.00E+00	0.8906	0.08584	1	0.6831	1	1	9.54E-01	1	0.9999	0.9998
	LC3	1.7		1	0.8496	1	1	0.002199	0.8335	0.06498	0.9707	0.9998	2.58E-01	0.9994	1	1
	LC4	2.177	0.5861		6.72E-01	0.9999	1	1.29E-03	6.51E-01	3.67E-02	8.78E-01	0.9944	1.55E-01	9.88E-01	1.00E+00	1
**Run2** (20°C, 100 l h^{-1})	LC1	0.9827	2.683	3.096		0.9838	4.48E-01	0.3662	1	0.977	1	1.00E+00	0.9999	0.9999	9.65E-01	9.48E-01
	LC2	1.026	0.6742	1.217	2.009		0.9989	1.09E-02	9.80E-01	2.13E-01	9.99E-01	1	5.71E-01	1.00E+00	1.00E+00	1
	LC3	2.555	0.8548	0.2135	3.538	1.529		0.000235	0.426	0.009874	0.716	0.9708	5.87E-02	0.9492	1	0.9999
	LC4	4.629	6.22	6.416	3.71	5.589	7.019		0.3854	0.9955	0.1793	4.01E-02	9.04E-01	0.0538	0.0113	0.005464
**Run3** (30°C, 25 l h^{-1})	LC1	1.027	2.728	3.138	0.04457	2.053	3.582	3.668		0.9809	1	1.00E+00	0.9999	0.9999	0.9586	0.9397
	LC2	3.073	4.773	5.051	2.09	4.099	5.628	1.755	2.045		0.8696	4.77E-01	1.00E+00	0.5542	0.1955	0.1308
	LC3	0.4491	2.149	2.597	0.5336	1.475	3.004	4.209	0.5782	2.624		1.00E+00	9.94E-01	1	0.9968	0.995
	LC4	0.4068	1.294	1.796	1.39	0.6194	2.148	5.01	1.434	3.48	0.8559		8.52E-01	1.00E+00	1.00E+00	1
**Run4** (30°C, 100 l h^{-1})	LC1	2.269	3.97	4.299	1.287	3.296	4.825	2.506	1.242	0.8033	1.82	2.676		8.99E-01	5.15E-01	4.21E-01
	LC2	0.2548	1.446	1.938	1.238	0.7713	2.3	4.867	1.282	3.328	0.7039	0.152	2.524		1	1
	LC3	1.281	0.31	0.8448	2.2	0.3207	1.11	5.572	2.242	4.155	1.701	0.9	3.403	1.042		1
	LC4	1.324	0.376	0.9377	2.307	0.2982	1.231	5.868	2.352	4.397	1.773	0.9176	3.594	1.07	0.04169	

Table 8 Supplementary Information. Results of ANOVA and Tukey's HSD testing with factors irradiance, temperature and flow on I_k in biofilms at the last sampling day. Tukey's Q below the diagonal, p value above the diagonal. Significant comparisons are red.

		**Run1** (20°C, 25 l h^{-1})				**Run2** (20°C, 100 l h^{-1})				**Run3** (30°C, 25 l h^{-1})			**Run4** (30°C, 100 l h^{-1})		
		LC1	LC2	LC3	LC4	LC1	LC2	LC3	LC4	LC1	LC2	LC3	LC1	LC2	LC3
**Run1** (20°C, 25 l h^{-1})	LC1		0.9997	0.02749	0.1192	1	2.75E-01	7.93E-03	0.9902	1	1	0.38	0.832	0.02154	0.1445
	LC2	1.269		0.001206	0.007512	1	0.02757	2.67E-04	1	0.9995	0.9995	0.06108	0.9993	0.000892	0.01064
	LC3	5.135	6.403		1	0.00194	0.9997	1	2.53E-04	0.03302	0.03329	1	2.06E-05	1	1
	LC4	4.393	5.695	0.8747		0.01163	1	0.9991	1.74E-03	0.1386	0.1396	1	0.000157	1	1
**Run2** (20°C, 100 l h^{-1})	LC1	1.091	0.1772	6.226	5.513		0.04025	4.43E-04	1	0.9999	0.9999	0.08294	0.9977	0.001444	0.0161
	LC2	3.865	5.133	1.27	0.4281	4.956		0.991	7.59E-03	0.3088	0.3104	1	0.000864	0.9994	1
	LC3	5.673	6.941	0.538	1.427	6.764	1.808		5.12E-05	0.009731	9.82E-03	0.9989	3.73E-06	1	0.9993
	LC4	1.826	0.5572	6.96	6.266	0.7344	5.691	7.498		9.85E-01	9.85E-01	2.13E-02	1.00E+00	1.83E-04	2.65E-03
**Run3** (30°C, 25 l h^{-1})	LC1	0.08474	1.353	5.05	4.306	1.176	3.78	5.588	1.911		1	0.4154	0.7992	0.02599	0.1663
	LC2	0.08859	1.357	5.046	4.302	1.18	3.776	5.584	1.914	0.003852		0.4171	0.7977	0.02621	0.1673
	LC3	3.616	4.751	0.9762	0.218	4.593	0.1596	1.457	5.249	3.541	3.537		0.003534	1	1
**Run4** (30°C, 100 l h^{-1})	LC1	2.66	1.392	7.795	7.123	1.569	6.525	8.333	0.8346	2.745	2.749	5.996		1.46E-05	0.000266
	LC2	5.245	6.514	0.1104	0.988	6.336	1.38	0.4276	7.071	5.16	5.156	1.075	7.905		1
	LC3	4.282	5.551	0.8526	0	5.373	0.4173	1.391	6.108	4.197	4.193	0.2136	6.942	0.963	

Table 9 Supplementary Information. Results of Spearman's rho-statistic applied to test the correlation between Chl *a* and AFDW.

Variable A	Variable B	rho	magnitude	p-Value
Chl *a* concentration	AFDW	0.8336164	0.6949163	1.58E-08
